# Pulse approach: a physics-guided machine learning model for thermal analysis in laser-based powder bed fusion of metals

**DOI:** 10.1007/s40964-024-00713-x

**Published:** 2024-07-26

**Authors:** Pooriya Scheel, Ehsan Hosseini

**Affiliations:** 1 Empa Swiss Federal Laboratories for Materials Science and Technology, Überlandstrasse 129, 8600 Dübendorf, Switzerland; 2 Department of Mechanical and Process Engineering, ETH Zürich, Leonhardstrasse 21, 8092 Zürich, Switzerland

**Keywords:** Thermal simulation, Laser powder bed fusion, Physics-guided machine learning, Feed-forward neural networks

## Abstract

Fast and accurate representation of heat transfer in laser powder-bed fusion of metals (PBF-LB/M) is essential for thermo-mechanical analyses. As an example, it benefits the detection of thermal hotspots at the design stage. While traditional physics-based numerical approaches such as the finite element (FE) method are applicable to a wide variety of problems, they are computationally too expensive for PBF-LB/M due to the space- and time-discretization requirements. Alternatives are sought to lower the computational burden of modelling this process and make part-scale simulations feasible, with machine learning (ML) techniques leading these efforts due to their exceptional flexibility and efficiency. Recently, there has been a growing trend towards integrating physical insights of the studied phenomena in ML workflows to improve their effectiveness. For the presented work, we hypothesized that the moving laser heat source could be treated as a sequence of pulses such that the solution to various scan patterns could be determined based on the thermal response to a laser pulse. First, a base function represented by a feed-forward neural network (FFNN) was proposed to establish the solution for laser scanning over a wide solid block. Next, inspired by the perturbation theory, a second FFNN was introduced to consider the impact of geometrical features on the temperature profiles as a correction to the base solution. The feasibility of training the pair of FFNNs within the proposed ‘pulse approach’ framework based on a few inexpensive FE simulations, and generalization to larger simulation domains are demonstrated. For a scan pattern not encountered during training, the paired networks are capable of accurately replicating the temperature profiles or history predictions of FE simulations in under one second, showcasing a considerable acceleration by orders of magnitude. The models and scripts used in this study are openly available in https://github.com/HighTempIntegrity/PIAM_Pulse2024.

## Introduction

Laser-based powder bed fusion of metals (PBF-LB/M) has become the most widespread metal additive manufacturing (MAM) technique [1, 2] owing to high design flexibility, good dimensional accuracy, low porosity, superior mechanical properties and good surface finish [1, 3, 4]. In PBF-LB/M, components are fabricated from thin layers of powder by selectively scanning them based on a 3D model using a high-intensity laser. The small size and high scanning speed of the laser heat source creates a highly transient temperature field with large localised gradients that move with the process-zone. These temperature profiles are one of the underlying factors in formation of high residual stresses and a unique microstructure that are particular to PBF-LB/M builds [5, 6]. Understanding the heat transfer behaviour during PBF-LB/M is the first step in optimising the process parameters, which can be possibly obtained using numerical models.

The physical phenomena involved in the process of exposing thin metal powder layers to a small, high-intensity laser heat source presents a challenging simulation problem. Various studies have focused on modelling the complex phenomena involved, such as the Marangoni effect, powder wetting, metal evaporation, and keyhole formation. These studies have successfully predicted outcomes like pore formation, balling behaviour, and rough surface finishes [7–9]. High-fidelity models are also particularly crucial for examining microstructure evolution in PBF-LB/M, yet the extensive level of discretization needed to accurately model these complex phenomena at a very small scale renders them computationally intensive [6, 7]. Recent advancements in high-fidelity modelling have leveraged graphics processing unit (GPU) compute power to expand the simulation domain from single tracks to multi-layer, thin-wall structures [9, 10]. This larger model has illustrated the cumulative evolution of wall morphology as various layers are deposited [9], highlighting the significance of enlarging the simulation domain.

A less computationally intensive approach involves the use of continuum-based finite element (FE) thermal models. These models do not account for certain physical aspects, such as the behaviour of individual powder particles, their interactions with the laser, each other, and the liquid metal, as well as the motion within the melt pool [11–13]. Despite these simplifications, such models are still pertinent for applications that are less sensitive to exact temperature profiles within the process zone, and yet require a fairly accurate representation outside of it, for instance, mechanical simulations aimed at predicting residual stress and distortion of PBF-LB/M parts [14–16]. Even with the lower simulation times due to simplifications, the fine discretisation requirement remains a challenge with these approaches [11]. One way to tackle this problem is to adopt adaptive re-meshing techniques to moderate the number of spatial degrees of freedom in continuum models by adaptively refining the mesh around the moving heat source [11, 17–19]. However, application to lengthy build jobs remains challenging due to the need for many small solution time increments. Alternatively, a common simplification for extending the simulations to the part-scale is the ‘lumped-heating’ approach, where the thermal energy of a few laser tracks or deposition layers is applied at once, hence much larger elements and solution increments can be used [20, 21]. While the resulting temperature field well represents the far-field behaviour, information about the process-zone temperature and effects of the scanning strategy are lost.

Alternative to physics-based simulations, data-driven techniques have been used for developing cheap numerical surrogate models for thermal analysis of PBF-LB/M. For instance, Anandan Kumar et al. [22] trained Gaussian processes (GP) based on FE simulations of a single track under a variety of laser powers and scanning speeds. Kizhakkinan et al. [23] similarly used a GP model to create a numerical surrogate based on CFD simulations for different laser powers. While these efforts showed good accuracy and computational performance, they were limited to a single track in scope, and relied on numerous FE/CFD simulations for training. Mozaffar et al. [24] trained a recurrent neural network (RNN) for predicting the temperature history in the direct energy deposition (DED) process, which could be extended to longer durations, but faced limitation for application to geometries dissimilar to the training data. Ren et al. [25] combined RNNs with deep neural networks (DNNs) to efficiently predict the thermal history in DED. While their approach could predict the temperature field for various cross-sections, it relied on 100 FE simulations lasting about 50 days for completion.

Outside the realm of heavily data-driven machine learning (ML) techniques, interest in applying physics informed neural networks (PINNs) to scientific problems has grown rapidly in recent years [26], since they can significantly reduce or even eliminate the need for large amounts of training data. Zhu et al. [27] and Hosseini et al. [28] used PINNs to model heat transfer for single laser tracks in PBF-LB/M with semi-supervised and unsupervised learning, respectively. However, it is imperative to extend the modelling domain far beyond single tracks in order to gain perspectives into temperature fields within parts during the PBF-LB/M process. Therefore, here we propose the pulse approach that uses the outcomes of only a few inexpensive FE simulations for training, and predicts temperature profiles for a multi-track scan pattern over multiple layers. This was achieved by treating the moving heat source as a series of very short laser scans, i.e. heat pulses, such that the overall temperature field could be approximated by considering the contribution of individual pulses.

A ‘base function’ corresponding to thermal response of a heat pulse was represented by a feed-forward neural network (FFNN) and trained based on inexpensive FE thermal simulation of e.g. a short (2 mm) laser track, as explained in Sect. 2. The trained network could accurately predict the temperature profiles of multiple long (8 mm) tracks. This base solution, however, needed a correction for considering the perturbations caused by geometrical features on the process-zone temperatures. Later in Sect. 3, we showcase how the effect of scanning near the edges of a small cuboid structure can be accounted for using a corrector function. It is important to note that at this stage, the temperature dependence of material parameters was neglected to constitute a linear heat transfer equation where superposition could be applied directly. In Sect. 4, the effect of adopting a simple temperature-dependent thermal material model on the performance of the pulse approach is presented. Finally, we discuss the computational benefits of the pulse approach in Sect. 5 and provide an outlook for further extension of this work. The details of FE thermal simulations that were conducted using the Abaqus software package is presented in appendix A, and the following focuses on the main topic of this study revolving the pulse approach as a surrogate model for such simulations.

## Forming the base solution

For modelling heat transfer in PBF-LB/M, the deposited thermal energy from a continuously moving heat source can be treated as a sequence of energy ‘pulses’, which follow the laser scan path. By combining the thermal response of each pulse, the transient temperature field of any scan pattern can be represented. In this section, we show that, in the absence of changing boundary conditions, e.g. scanning over a semi-infinite space, a single ‘base function’ can represent the thermal solution (for a given set of PBF-LB/M process parameters and material properties).

### Conceptualization

In response to a laser pulse, i.e. a very short scan length, the temperature field *T*(*x*, *y*, *z*, *t*) involves a sharp increase in temperatures, which is quickly dissipated by conduction. This can be represented in terms of the base transient thermal energy field *u*(*x*, *y*, *z*, *t*) via the following relationship:

1 $$\begin{aligned} T(x,y,z,t) = T_0 + \frac{u(x,y,z,t)}{\rho c_p} \end{aligned}$$

Here, the spatial coordinates $[x,y,z]^T$ denote any point in the global Cartesian coordinate system, and *t* is the total time passed since the start of laser scanning. Furthermore, $\rho$ is the density, $c_p$ is the specific heat capacity, and $T_0=25^{\circ }\text {C}$ is the initial temperature of the modelling domain.

In the absence of changes to the boundary conditions, temperature-independent material properties, and treating the laser heat source as a sequence of pulses, the calculated energy field for a laser track can be stated in terms of a summation over contributions of pulse events as:

2 $$\begin{aligned} u_{\text {FE}}(x,y,z,t) \approx \sum _{i=1}^{N} u_{b,i}(x-x_{p,i},y-y_{p,i},z-z_{p,i},t-t_{p,i}) \end{aligned}$$

**Fig. 1:**
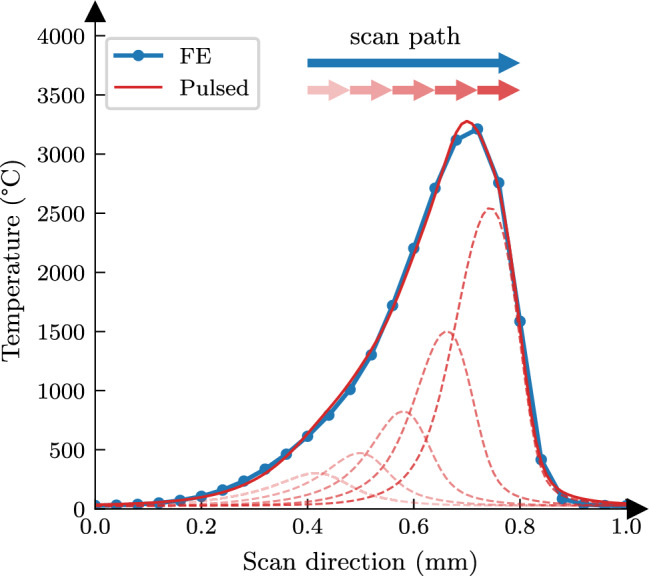
Visualisation of the framework outlined by Eq. (2), highlighting the superposition of pulsed thermal responses for representing a moving heat source. The solid red line is the result of summing the dashed pulse responses, which closely matches FE predictions for a short laser track

Here, $u_{\text {FE}}$ is the thermal solution to the moving heat source (e.g., calculated by the FE simulation), $u_{b,i}$ is the thermal energy field induced by the *i*-th laser pulse, and the summation is applied over a total of *N* pulses that are required to represent the scanned laser path. The spatial coordinates $[x_{p,i},y_{p,i},z_{p,i}]^T$ represent the centre of a laser scan segment corresponding to *i*-th pulse event, and $t_{p,i}$ is the time at which the laser heat source reaches the centre of a pulse event. For any time *t*, the summation is evaluated only over the active pulses such that $t>t_{p,i}$. A visual representation of this relationship is provided in Fig. 1. As seen in dashed curves, the contribution of each pulse to the overall temperature profile decays with time.

More generally, the changes in laser movement direction for a multi-track scan pattern are considered by applying a rotation to align the local X-axis with the scan direction. Therefore, Eq. (2) can be expressed as:

3 $$\begin{aligned} u_{\text {FE}}(x,y,z,t) \approx \sum _{i=1}^{N} u_{b,i}(x_{l,i},y_{l,i},z_{l,i},t_{l,i}) \end{aligned}$$

**Fig. 2:**
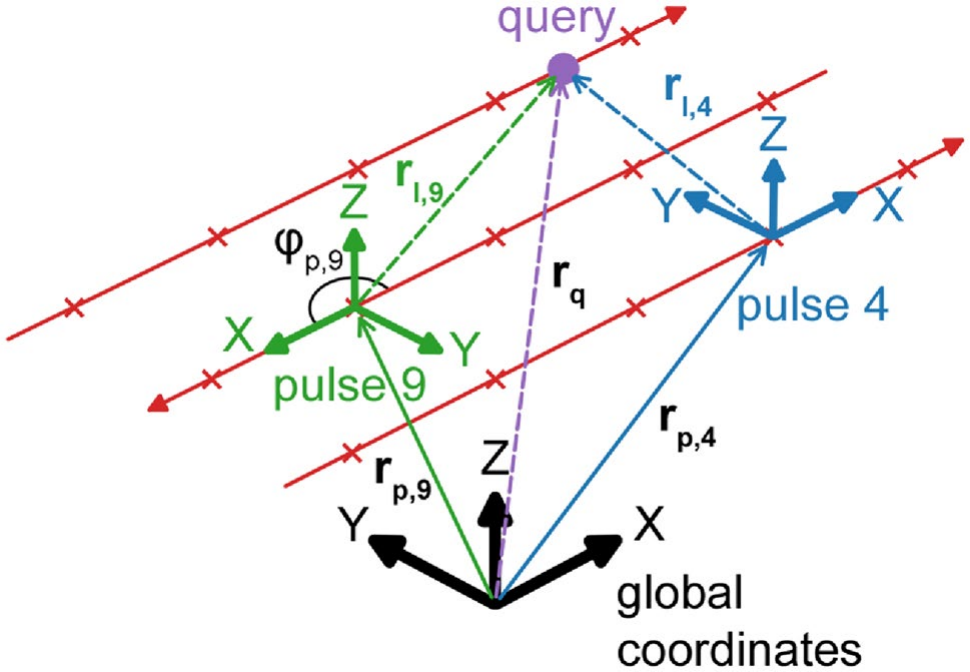
The relationship between local coordinate system of pulse events and the global coordinate system. The $\varvec{r}$ symbols denote $[x,y,z]^T$ spatial vectors between different points

In above equation, the local coordinates $[x_{l,i},y_{l,i},z_{l,i}]^T$ and the relative time $t_{l,i}$ are related to the global coordinate system and total time via:

4 $$\begin{aligned} \begin{bmatrix} x_{l,i}\\ y_{l,i}\\ z_{l,i}\\ t_{l,i} \end{bmatrix} = \begin{bmatrix} \cos (\phi _{p,i}) &{} \sin (\phi _{p,i}) &{} 0&{} 0\\ -\sin (\phi _{p,i}) &{} \cos (\phi _{p,i}) &{} 0&{} 0\\ 0 &{} 0 &{} 1&{} 0\\ 0 &{} 0 &{} 0&{} 1\\ \end{bmatrix} \times \begin{bmatrix} x-x_{p,i}\\ y-y_{p,i}\\ z-z_{p,i}\\ t-t_{p,i} \end{bmatrix} \end{aligned}$$

where $\phi _{p,i}$ is the rotation angle between the laser scan direction and the global X-axis. This relationship is illustrated in Fig. 2.

In summary, Eq. (3) shows that the function $u_{b}$ remains the same for every pulse event, and the response to various laser scan patterns can be acquired by superposing this function with appropriate consideration of time and space translations. Next, the base solution $u_b$ is represented by a fully connected FFNN with *L* hidden layers as:

5 $$\begin{aligned} u_{b}(x_l,y_l,z_l,t_l;\varvec{\theta }_b)& = (\sigma _o \circ C_L \circ \dots \sigma _h \nonumber \\{} & {} \circ C_{1} \circ \sigma _h \circ C_{0})(x_l,y_l,z_l,t_l) \end{aligned}$$

In above equation, $\circ$ denotes function composition such that in the *j*-th layer, an affine transformation $C_j$ is applied to an input vector $\varvec{z}_j$ to provide $C_j(\varvec{z}_j)=W_j z_j+b_j$. Parameter $\sigma _h$ denotes a non-linear activation function that is applied between consecutive affine transformations. An additional non-negative filter $\sigma _o(x)$ was added on the output layer based on the physical knowledge that the network output, i.e., the added thermal energy due to laser exposure, can only take positive values ($u_b\ge 0$). Finally, $\varvec{\theta }_b$ denotes the collective weights and biases defining the base neural network, also referred to as network parameters.

Inspired by [28], a feed-forward neural network, with 6 hidden layers containing 24 neurons each, consistently demonstrated fast convergence across various ensemble trainings. However, instead of the sinusoidal activation function that works best for constructing a PINN solution for the quasi-steady-state thermal response in [28], the tangent hyperbolic function proved to perform better in representing the long-term transient temperature profiles in the current study. That is:

6 $$\begin{aligned} \sigma _h(x)=\tanh (x) \end{aligned}$$

For the output filter, a softplus function showed the best convergence stability defined as the following:

7 $$\begin{aligned} \sigma _o(x) = \ln (1+\exp (x)) \end{aligned}$$

Furthermore, to ensure that the network satisfies the aforementioned symmetry in Y direction, the input Y-coordinate was passed through an absolute value filter, and $\partial _{y}u_b|_{y=0}=0$ was enforced in the loss function as discussed next.

### Defining the loss terms

In the proposed scheme, the FE-predicted temperature histories were used to establish the ground truth, with more details of the simulation setup provided in appendix A. The main aim of the training process is to tune network parameters such that the target temperature field would be closely approximated by the base function in Eq. (3). Accordingly, the first loss term is defined as the mean squared error (MSE) of the superposed network predictions and the FE results:

8 $$\begin{aligned} \mathcal {L}_{\text {FE}}(\varvec{\theta }_b) = \text {MSE}\left( (T_{\text {FE}}-T_0)\rho c_p - \sum _{i=1}^{N} u_{b,i}(\varvec{\theta }_b)\right) \end{aligned}$$

Here, the summation is applied over *N* pulse events representing the scan pattern used to generate the training data, and MSE is applied over a subset of temperature points chosen randomly across space and time coordinates.

Furthermore, to ensure that the previously mentioned requirement for $\partial _{y}u_b|_{y=0}=0$ is satisfied, an additional loss term was defined such that:

9 $$\begin{aligned} \mathcal {L}_{\text {sym}}(\varvec{\theta }_b) = \text {MSE}\left( \left. \frac{\partial u_b(\varvec{\theta }_b)}{\partial y}\right| _{y=0}\right) \end{aligned}$$

Using automatic differentiation, the partial derivative of the output with respect to the input local Y-coordinate could be effortlessly calculated. By combining Eqs. (8) and (9), the total loss function was defined as:

10 $$\begin{aligned} \mathcal {L}_b(\varvec{\theta }_b) = \mathcal {L}_{\text {FE}}(\varvec{\theta }_b) + \lambda _{\text {sym}} \mathcal {L}_{\text {sym}}(\varvec{\theta }_b) \end{aligned}$$

$\lambda _{\text {sym}}$ was manually tuned in the [1,16] range, where it was found that $\lambda _{\text {sym}}=4$ ensures that all loss terms contribute similarly. Through minimizing the loss function by tuning the network parameters, a specific set of parameters $\varvec{\theta }^*_b$ can be found such that:

11 $$\begin{aligned} \varvec{\theta }^*_b = \operatorname*{arg\,min}_{\varvec{\theta}_b}\mathcal {L}(\varvec{\theta }_b) \end{aligned}$$

Finally, the optimised network parameters $\varvec{\theta }^*_b$ would constitute a trained variant of $u_b$ that can be used to determine the thermal response to various scan patterns. In the following, the trained neural network corresponding to the base thermal response is denoted as:

12 $$\begin{aligned} u^*_b = (x_l,y_l,z_l,t_l;\,\,\varvec{\theta }^*_b) \end{aligned}$$

The presented framework was implemented in Python 3 using the open-source PyTorch library, where the limited-memory BFGS optimiser was used for Eq. (11).

### Training the base function

In choosing the scan pattern for defining the training datasets, anything from a single laser pulse to bidirectional multi-tracks is possible. To examine the effect of different training datasets on prediction accuracy, the base network was separately trained with FE simulation data of a single ${80}\,{\upmu }\text {m}$ pulse, one 2 mm scanned track, and 7$\times$2 mm bidirectional laser tracks. The trained network variants are labelled $u^*_{b1}$, $u^*_{b2}$, and $u^*_{b3}$, respectively.

**Fig. 3:**
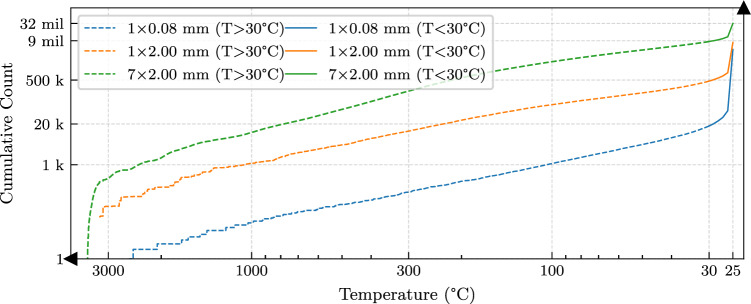
Distribution of training data generated from three FE simulations used to train the base function

By representing each space-time coordinate and the corresponding temperature value $T_{\text {FE}}$ as a data-point, a very large dataset can be extracted from a single FE simulation. However, most of this data corresponds to low temperature values in regions far from the process-zone or during the cooling phase. The bias in the population distribution can be seen in the descending cumulative count of temperatures plotted in Fig. 3 for the three FE simulations. In order to ensure a balanced sampling strategy between low/high temperatures during training, the total data was divided into two portions; a high temperature set containing all of the process-zone temperatures, and a low temperature set for the far-field region. Accordingly, the total FE loss was calculated as the sum of losses over the two sample sets:

13 $$\begin{aligned} \mathcal {L}_{\text {FE}} = \mathcal {L}_{\text {FE}}(T_{\text {FE}}>30^{\circ }\text {C})+\mathcal {L}_{\text {FE}}(T_{\text {FE}}<30^{\circ }\text {C}) \end{aligned}$$

A small portion of the data was set aside for validation, and the remainder was used for the training process. As the amount of data remained relatively large, especially in the low-temperature group, it was necessary to divide the training set into smaller batches, and use a limited number of them before reshuffling the batches between different training epochs. A detailed summary of the split, training times and the number of pulse events in each scenario is provided in Table 1. All presented neural networks in this work were trained on a single CUDA-enabled computational node equipped with a GeForce RTX 3090 graphics processing unit.

**Table 1:** Overview of training information for different variants of the base function

Variant		$u^*_{b1}$	$u^*_{b2}$	$u^*_{b3}$
Scan size (mm)		0.08 mm	1$\times$ 2 mm	7$\times$ 2 mm
Pulse events (–)		1	25	175
Total points		4’683’735	7’895’439	31’983’219
Training batch size	$T_{\text {FE}}>30^{\circ }\text {C}$	13’742	180’000	105’000
	$T_{\text {FE}}<30^{\circ }\text {C}$	300’000	300’000	105’000
Validation size	$T_{\text {FE}}>30^{\circ }\text {C}$	3’436	92’720	105’000
	$T_{\text {FE}}<30^{\circ }\text {C}$	300’000	300’000	105’000
Batches per epoch		1	2	4
Training time (min)		26	174	603
Iterations (–)		$2.5\,\times 10^{4}$	$5\,\times 10^{4}$	$1\,\times 10^{5}$

**Fig. 4:**
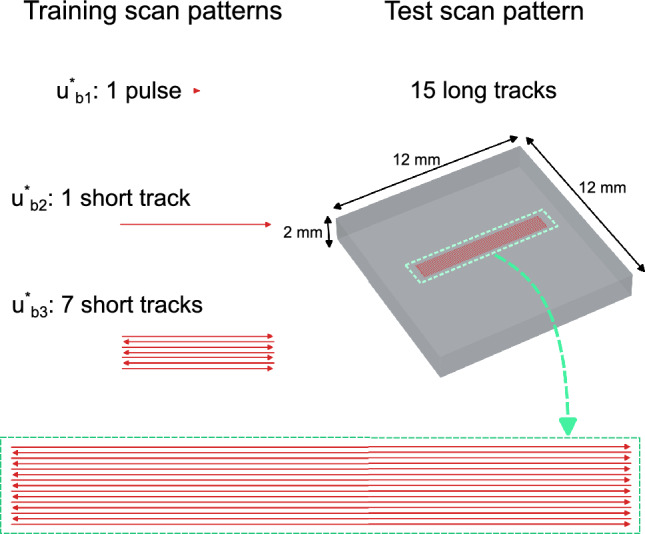
Comparison of scan patterns used in FE simulations for training and testing the base function

For testing the prediction accuracy of the base function variants, a separate reference FE simulation for a ${1\,\text {mm}\,\times \,8\,}\text {mm}$ scan area (consisting of 15$\times$8 mm bidirectional laser tracks) was employed. The scan patterns used for training and testing of the networks are visually compared in Fig. 4. It should be noted that the calculated process zone temperatures significantly exceed expectations due to the adoption of a simplified material model that does not account for the latent heat of fusion. Nevertheless, these results can be used to numerically verify the accuracy of network predictions against FE heat transfer models of the PBF-LB/M process, as discussed in the following.

**Fig. 5:**
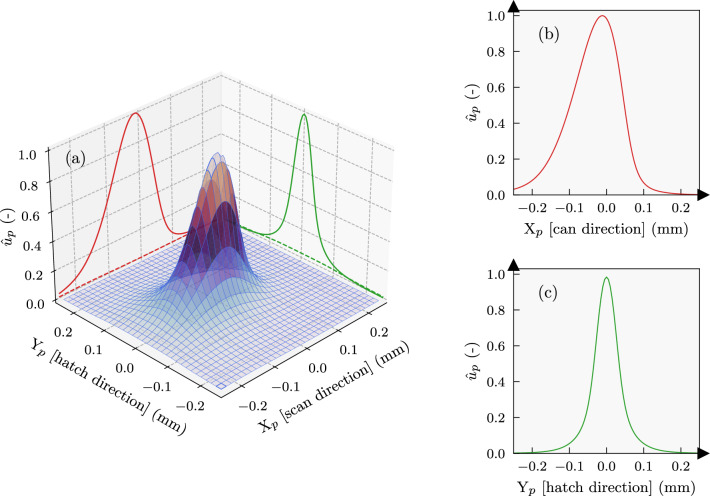
The normalised thermal energy distribution ${\hat{u}}_p$ formed immediately after exposure to a short laser scan corresponding to a single pulse. (a) Three-dimensional profile in the scanning plane. (b) Skewed profile along the scan direction. (c) Symmetric profile in hatch direction. These profiles were generated using the trained base network

### Base function results

**Fig. 6:**
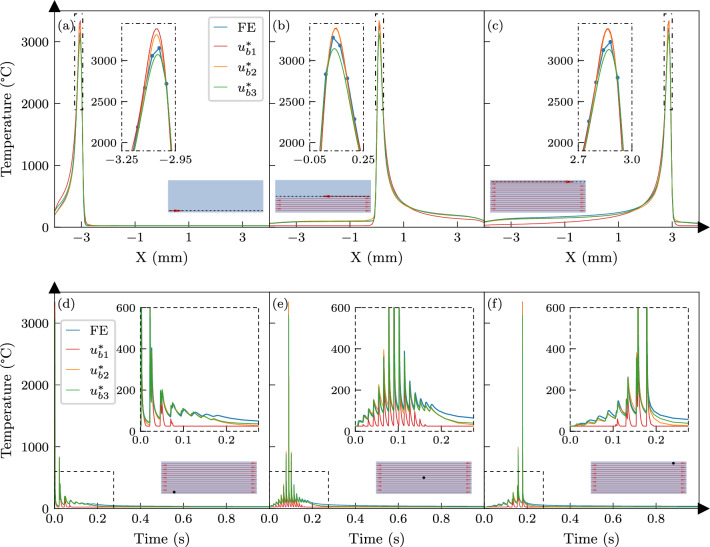
Comparison of temperature predictions from three base functions with FE simulation results for 15 long laser tracks. **a**, **b**, **c** The temperature distribution along three different tracks denoted by the dashed line in the scan area schematics. The insets zoom on the peak temperatures in the process-zone. **d**, **e**, **f** The temperature histories at three different locations denoted by the black dots in the scan area schematics. The insets focus on the dashed box to highlight the accuracy gains for training on a larger scan area

To illustrate the shape of a trained base function, Fig. 5 shows it in normalised form over the scanning plane, and its projections along the scan and hatch direction. It is interesting to note that while the symmetry condition along the hatch direction Y is well satisfied, the distribution is slightly skewed in scan direction X due to the movement of the laser over the span of a scan pulse. By superposing the pulsed responses according to Eq. (3) for 15$\times$8 mm bidirectional tracks, the network response can be tested against an equivalent reference FE simulation following the setup described in appendix A. Temperature distributions and histories at the first, middle, and last laser tracks of the reference model are compared with network predictions in Fig. 6. The zoomed insets in Fig. 6a–c show that all three network variants closely matched the maximum observed temperatures in the FE simulation. However, the $u^*_{b1}$ base network underpredicted the temperature distribution along the laser trail, while $u^*_{b2}$ and $u^*_{b3}$ showed lower errors. This suggests that better representation of longer laser scanning in the training datasets of $u^*_{b2}$ and $u^*_{b3}$ contributed to their improved performance.

The thermal response in few milliseconds immediately after laser exposure and an overview of thermal behaviour over a longer period are provided in Fig. 6a–c and Fig. 6d–f, respectively. The underestimations by the $u^*_{b1}$ solution are again evident, and the differences between $u^*_{b2}$ and $u^*_{b3}$ are more pronounced. The inclusion of simulation data from a laser track in the training significantly improved the network accuracy over time in the $u^*_{b2}$ variant, and the incorporation of data from a multi-track scan further enhanced the results in $u^*_{b3}$.

In summary, all network variants accurately represented the temperature profiles in the process-zone, but using a larger training dataset greatly improved the long-term predictions. It is worth noting that at no stage in the training process were the networks exposed to the thermal behaviour of an 8 mm scan length, which indicates that the base function could well predict the temperature profiles for scan patterns larger than the training dataset.

The average error of the trained models over time can be quantitatively represented by the mean absolute percentage error (MAPE) defined as:

14 $$\begin{aligned} \text {MAPE} = \frac{100}{n} \sum _{t=1}^{n}\left| \frac{T_{\text {NN}}(t)-T_{\text {FE}}(t)}{T_{\text {FE}}(t)}\right| \end{aligned}$$

The above sum is applied over *n* time-frames used to solve the reference FE simulation. Additionally, the percentage error in maximum temperature prediction of the network for any given point was defined as:

15 $$\begin{aligned} \text {T}_{max}\text {PE} = 100\times \left| \frac{T_{\text {NN},max}-T_{\text {FE},max}}{T_{\text {FE},max}}\right| \end{aligned}$$

**Fig. 7:**
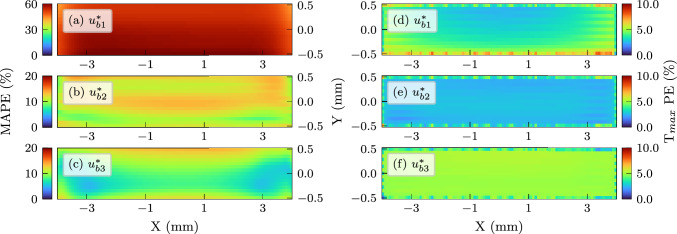
Mean absolute errors (MAPE) and peak temperature errors ($\hbox {T}_{max}$PE) over the scanned tracks for three base networks in terms of percentage. **a**, **b**, **c** MAPE for $u^*_{b1}$, $u^*_{b2}$ and $u^*_{b3}$, respectively. **d**, **e**, **f** $\hbox {T}_{max}$PE for $u^*_{b1}$, $u^*_{b2}$ and $u^*_{b3}$, respectively. It should be noted that the scale of the colour map in (a) was increased to better highlight the larger prediction errors of this variant

Distribution of the two error indices over the laser scan path in the reference modelling domain (15$\times$8 mm tracks) are plotted in Fig. 7 as colour maps. As previously seen in time history comparisons, $u^*_{b1}$ largely deviates from the reference solution over time, but training with larger scan areas in $u^*_{b2}$ and $u^*_{b3}$ considerably improves the results. In case of percentage errors in peak temperatures, the accuracy of the base networks is relatively consistent. This shows potential benefits of this approach for applications focusing on peak temperatures, e.g. hotspot detection, since training on a small FE model would suffice. If a more accurate representation of time histories and temperature profiles is needed, the network should be trained on a slightly larger scan area.

## Effect of geometry

The base function was developed to represent the thermal response in PBF-LB/M for situations where the effect of system boundary is negligible, e.g. scanning in the middle of a large solid block. However, laser scanning at the edges of a part or at heights far from the substrate would result in development of a slightly different thermal response. This brings about perturbations with respect to base function predictions, as a response to the changing boundaries around the moving heat source. By parametrising this effect in terms of boundary distances, a correction to the base function can be defined to account for the thermal changes. The feasibility of this approach to account for the changes in thermal response during scanning a simple $8\,\text {mm}\,\times \,1\,\text {mm}\,\times \,1\,\text {mm}$ cuboid geometry consisting of 33 layers is demonstrated. The details of the FE simulations for generating the training data and testing the performance of the trained network are provided in appendix A. The following focuses on problem formulation, training, and performance of the geometry correction function.

### Defining the pulse corrector

The temperature profiles for scanning at higher build-heights and closer to the boundaries are underestimated by the base function which represents maximum heat loss due to conduction. This increase is dependent on the distance from the boundaries and the build plate. To account for this effect in the proposed pulsed approach, a corrector function $u_{c}$ can be defined to extend the relationship established in Eq. (3):

16 $$\begin{aligned} u_{\text {FE}} \approx \sum _{i=1}^{N}\left( u_{b,i} + u_{c,i} \right) \end{aligned}$$

The purpose of $u_{c}$ is to determine the correction to temperature distributions from the base solution as a function of the pulse distance to the boundaries in the scan direction ($d_{s}$), hatch direction ($d_{h}$), and the build plate ($d_{b}$). Thus, it can be formulated as:

17 $$\begin{aligned} u_{c,i} = u_{c,i}(x_{l,i}, y_{l,i}, z_{l,i}, t_{l,i}, d_{s,i}, d_{h,i}, d_{b,i}) \end{aligned}$$

It should be noted that the input distance parameters $[d_{s,i}, d_{h,i}, d_{b,i}]^T$ remain constant regardless of which space-time input coordinates $[x_{l,i}, y_{l,i}, z_{l,i}, t_{l,i}]^T$ the function is evaluated for. The corrector function $u_{c}$ was represented by a FFNN similar to the base function:

18 $$\begin{aligned}&u_{c}(x_l,y_l,z_l,{t}_{l},d_{s},d_{h},d_{b};\varvec{\theta }_c) \nonumber \\&\quad = (\sigma _o \circ C_L \circ \dots \sigma _h \circ C_{0})(x_l,y_l,z_l,{t}_{l},d_{s},d_{h},d_{b}) \end{aligned}$$

Since the pulse corrector mainly supports the base function, it was expected to be representable by a similar network architecture. Therefore, the same tangent hyperbolic activation function $\sigma _h(x)=\tanh (x)$, and a softplus filter $\sigma _o(x) = \ln (1+\exp (x))$ on the output were chosen. Albeit, the less complexity involved in correcting for an already existing base solution points towards the possibility of reducing the network size. Accordingly, diminishing returns were observed in increasing the network depth beyond 3 layers, and thus, an FFNN with 3 hidden layers and 24 nodes per layer was used to represent the pulse corrector function.

### Training for geometry effects

To train $u_c$, the base function was first evaluated for the modelling domain and subtracted from the FE solution to determine the required correction at various locations in the geometry. Hence, the loss function of $u_c$ was defined as the following:

19 $$\begin{aligned} \mathcal {L}_{\text {FE}}(\varvec{\theta }_c) = \text {MSE}\left( \left( (T_{\text {FE}}-T_0)\rho c_p - \sum _{i=1}^{N} u^*_{b,i} \right) - \sum _{i=1}^{N} u_{c,i}(\varvec{\theta }_c) \right) \end{aligned}$$

Here, $\varvec{\theta }_c$ denotes the network parameters of the corrector function. It is important to devise the training dataset in a way that all scenarios during PBF-LB/M of the target part are well represented by a limited number of FE models. This was achieved by exploring physical insights about the thermal response in PBF-LB/M of the studied structure. For instance, identical heat transfer conditions in four quarters of the rectangular cross-section implied that directionality is unimportant for the distance parameters, and capturing the behaviour of one quarter is sufficient. Furthermore, $d_{s}$ only becomes relevant when the laser scans close to the corners of the cuboid. This allowed us to place an upper limit of 0.5 mm on $d_{s}$ such that the behaviour remained consistent for the inner portion of the scan tracks, i.e. $u_c(d_s>0.5)=u_c(d_s=0.5)$. Overall, FE simulations for ${0.5\,\text {mm}\,\times \,0.5}\,\text {mm}$ scan areas in the middle and corners of a ${4\,\text {mm}\,\times \,1\,\text {mm}\,\times \,1}\,\text {mm}$ cuboid were conducted to establish the training dataset (further information is provided in appendix A).

In the proposed training scheme, the corrector function is trained after the base function, which makes it possible to reach at a different corrector depending on which base solution is chosen. In the following, correctors for the best performing base function in the previous section $u^*_{b3}$, and the easy-to-constitute $u^*_{b1}$ are evaluated and compared with reference FE simulations.

### Error of network pairs

**Fig. 8:**
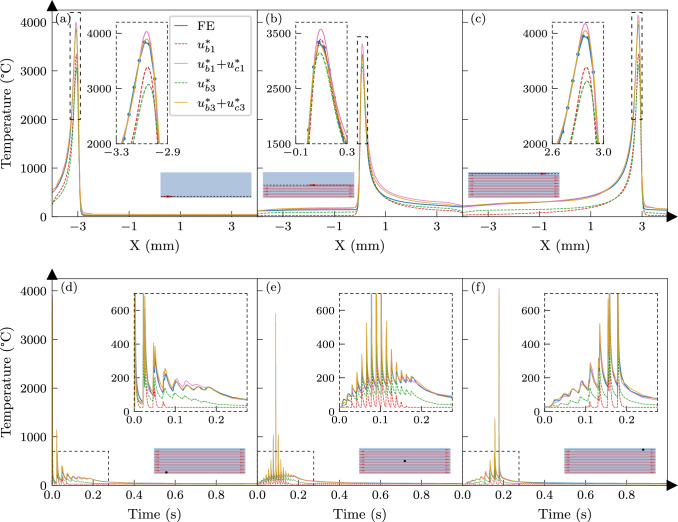
Temperature predictions from two network pairs with and without the correction compared to the FE response for PBF-LB/M of the 17th layer in the $8\,\text {mm}\,\times \,1\text {mm}\,\times \,1\,\text {mm}$ cuboid. **a**, **b**, **c** The temperature distributions along three different tracks denoted by the dashed line in the scan area schematics. The insets focus on the peak temperatures in the process-zone. **d**, **e**, **f** The temperature histories at three different locations denoted by the black dot in the scan area schematics. The insets zoom on the dashed box

The trained correctors were summed up with their base functions to evaluate their effectiveness in predicting the FE thermal simulation for PBF-LB/M of entire layers of the cuboid involving 15$\times$8 mm bidirectional laser tracks. To showcase the results, the temperature histories and distributions along the scan path for the first, middle and last tracks of layer 17 are plotted in Fig. 8. Looking at temperature distributions along different laser tracks in Fig. 8a–c, a large deviation between the base solution and the reference FE simulation on the edges is observed. Here, the correctors managed to effectively account for the required temperature increase to the base solution for closely approximating the FE response. In Fig. 8d–f, both correctors accounted for the increased temperature levels due to lower heat dissipation at the increased build height. However, $u^*_{b3}$+$u^*_{c3}$ provided smoother temperature profiles than $u^*_{b1}$+$u^*_{c1}$ since its base solution could better predict the thermal response of multi-track laser scanning.

**Fig. 9:**
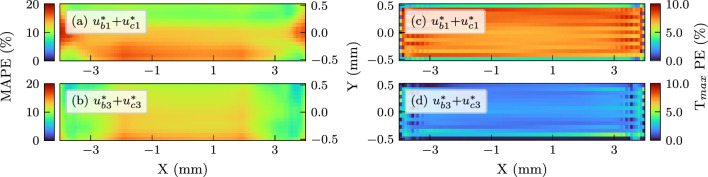
MAPE and peak temperature errors on layer 17 based on two network pairs. **a**, **b** the mean absolute percentage error for the $u^*_{b1}+u^*_{c1}$ and $u^*_{b3}+u^*_{c3}$ networks, respectively. **c**, **d** Absolute percentage error in $\hbox {T}_{max}$ for the $u^*_{b1}+u^*_{c1}$ and $u^*_{b3}+u^*_{c3}$ networks, respectively

Average network performance in terms of MAPE and peak temperature errors for layer 17 are presented in Fig. 9. Similar to the behaviour observed while comparing the temperature histories, adding the correction closely matches the FE thermal response as seen in MAPE values in Fig. 9a and b. In case of peak temperature predictions, the combined response manages to provide relatively accurate results for the whole domain as shown in Fig. 9c and d. Particularly, better performance was observed for $u^*_{b3}$+$u^*_{c3}$. It should however be noted that the proposed implementation of the corrector function can only account for scanning over a cuboid structure. More complex geometrical features such as overhangs and support structures require a more robust parametrisation.

## Effect of temperature dependent properties

**Fig. 10:**
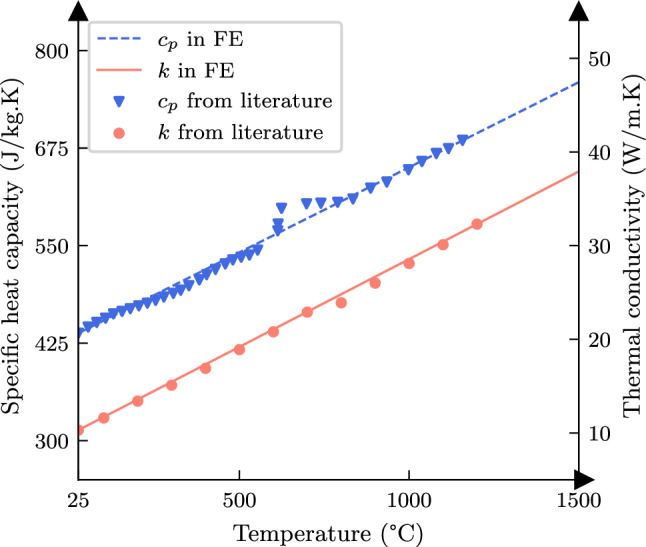
Temperature-dependent thermal properties of Hastelloy X based on [29]

So far, the pulse approach was demonstrated to work well in representing the solution of the linear heat transfer equation, i.e. for consideration of temperature-independent material properties, where the principle of superposition can be applied directly. However, material’s thermal properties, such as specific heat capacity and thermal conductivity, are known to vary with temperature, as shown for Hastelloy X in Fig. 10. Consideration of these variations gives rise to a non-linear heat transfer equation where the superposition of pulse solution as proposed in Sect. 2.1 does not hold strictly. In order to investigate the deviation of the proposed pulse approach for temperature-dependent properties with respect to the FE solution, the temperature dependence of specific heat capacity and thermal conductivity of Hastelloy X were considered and used to conduct FE simulations for the $u^*_{b3}$ scan pattern and the reference model as previously shown in Fig. 4. A similar training campaign was then carried out for the base function using the new data with the differences mentioned in the following.

For temperature-dependent specific heat capacity $c_p(T)$, the temperature–energy transformation outlined in Eq. (1) can be written in the general form as:

20 $$\begin{aligned} u = \int _{T_0}^{T}{\rho c_{p}(T)} \, \text {d}T \end{aligned}$$

Assuming $c_p(T)=c_1T+c_0$, the integration can be solved for *u* such that:

21 $$\begin{aligned} u = \rho \left( \frac{c_1}{2}(T^2-T_0^2)+c_0(T-T_0)\right) \end{aligned}$$

By inverting this relationship, we can write *T* as:

22 $$\begin{aligned} T = \sqrt{\frac{2u}{\rho c_1}+\left( T_0+\frac{c_0}{c_1}\right) ^2}-\frac{c_0}{c_1} \end{aligned}$$

This constitutes a variant of Eq. (1) for linear temperature-dependence of specific heat capacity. Accordingly, Eq. (8) can be rewritten as:

23 $$\begin{aligned} \mathcal {L}_{\text {FE}}(\varvec{\theta }_b) = \text {MSE}\left( \rho \left( \frac{c_1}{2}(T_{\text {FE}}^2-T_0^2)+c_0(T_{\text {FE}}-T_0)\right) - \sum _{i=1}^{N} u_{b,i}(\varvec{\theta }_b)\right) \end{aligned}$$

The above function was used to train a new variant of the base function (referred to as $u^{*\prime }_b$) using the same network architecture and total loss function as before.

**Fig. 11:**
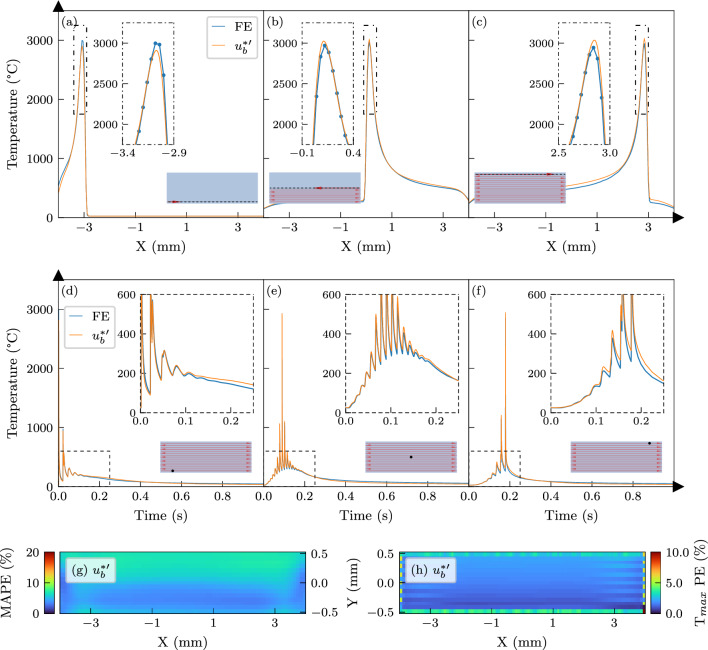
Accuracy of the base function that was trained and tested using the results of temperature-dependent FE simulations. **a**, **b**, **c** The temperature distributions along three different tracks denoted by the dashed line in the scan area schematics. The insets focus on the peak temperatures in the process-zone. **d**, **e**, **f** The temperature histories at three different locations denoted by the black dots in the scan area schematics. **g**, **h** The mean absolute error (MAPE) and peak temperature error ($\hbox {T}_{max}$PE) distributions over the scanned area, respectively

In Fig. 11a–c, the temperature distributions across three different tracks are compared with the FE solution, indicating overall good performance. The insets, which focus on peak temperatures, reveal that the trained network finds an ‘in-between’ solution that slightly underestimates the temperature response initially, but tends towards overestimation in later tracks. Figure 11d–f compares the predicted temperature history of $u^{*\prime }_b$ with the FE reference model at three different spots. A closer inspection of the discrepancies highlighted in the insets of these figures shows that the network tends to overpredict the FE results over a longer duration. This behaviour can be attributed to the effect of temperature-dependent properties on the thermal response over extended periods. As the material heats up, its thermal conductivity increases, facilitating greater heat dissipation. This leads to lower temperature values than those predicted by the network, which is based on the smaller scan areas used in its training. This trend is also evident in the error maps shown in Fig. 11g–h, where accuracy diminishes for the upper half of the rectangle, corresponding to laser scanning over preheated material. Nevertheless, as long as the temperature dependence of the material properties is moderate, the proposed approach yields accurate results. To more accurately represent this behaviour, further development of the base function is necessary.

## Discussion

### Computational performance

The results presented so far demonstrated that integrating physical information into a neural network, and training it based on data from a limited number of FE simulations involving small laser scans can be used to form a solution for thermal analysis of PBF-LB/M with extensibility to longer multi-track scan patterns. The approach involved a superposable base function to represent the temperature profiles away from the boundaries of the model, and a corrector function which accounted for variations in the solution from laser scanning near the edges or various build heights. The primary objective in seeking this solution was to decrease the time required for heat transfer modelling in the PBF-LB/M process. However, comparing the computational resources needed for a machine learning approach and a finite element solution can be challenging due to hardware discrepancies. Artificial neural networks are typically trained and evaluated on graphics processing units (GPU), while most FE solvers rely on central processing units (CPU) for computations. Nevertheless, comparisons can be made with respect to network evaluation times versus the FE simulation time required to reach similar results.

Evaluating the network outcomes presented in Fig. 8 took only 0.7 and 0.6 s for the time histories and temperature distributions, respectively. For generating the error maps shown in Fig. 9, more evaluations were required. However, with GPU parallelisation, it was possible to generate temperature histories for all 1500 coordinates in just 1.9 min. The linear FE reference solution used in the comparisons took 200 min to solve an entire layer before the results could be extracted. Adopting temperature-dependent thermal properties in Sect. 4 further increased the calculation time up to 937 min. A summary of these results is provided in Table 2. Although replicating the full outcome of an FE model through network evaluations can be time-consuming, it is often the case that only specific aspects of the thermal response, such as peak temperatures or process-zone contours, are of interest. In such scenarios, the pulse approach provides significant computational advantages. Low evaluation times offer great benefits in areas like process control, where quick and reliable information about peak temperatures in the process-zone can be utilised to adjust the laser power during the fabrication process. Furthermore, FE solutions for larger models and a longer scan duration require substantial disk space for storage. In contrast, the network parameters in the pulse approach occupy only a few kilobytes, and the necessary temperature profiles can be evaluated on-demand.

**Table 2:** Comparison of temperature history evaluation time of FFNNs versus corresponding FE simulations for one 15$\times$8 mm scanned layer

Approach	Domain size	Material model	Duration (min)
Base+corrector networks	1 point	–	0.01
	1 layer	–	1.9
Reference FE simulation	Full	Constant	200
	Full	Temperature-dependent	937

### Outlook

In order to further extend the pulse approach, it is crucial to first recognise its limitations. The experimental relevance of network predictions, serving as a surrogate model, is significantly dependent on the FE modelling assumptions, which form the foundation for training. This means that the strategy is bound by the inherent limitations of continuum thermal FE modelling, such as ignoring phenomena related to liquid motion, including the Marangoni effect and powder wetting. However, it is possible to refine the assumptions used in training data, leading to a more advanced variant of neural networks, as exemplified in Sect. 4 with the implementation of temperature-dependent material considerations. There, we observed that a static base function does not account for increased heat conduction at higher temperature levels, necessitating adjustments to the network architecture to address this effect. A potential solution could involve incorporating information about adjacent pulse events as inputs to the network, thus introducing an ‘awareness’ of the scan path and the impact of heat accumulation on temperature profiles. Additionally, integrating the latent heat of fusion could be achieved by compensating for the increased non-linearity in specific heat capacity, as outlined in Eq. (20).

Another limitation of the presented approach lies in the corrector function that is only suited for a 1 mm wide cuboid structure. Given the prevalence of manufacturing complex geometries like holes, overhangs, and support structures in PBF-LB/M, a more generalized parametrisation of these effects on thermal response is necessary. Although improving the distance parametrisation outlined in Sect. 3, is one solution, a potentially more effective method could involve training a complex neural network directly on the thermal response of laser scanning over varied shapes, eliminating the need to divide the solution into base and corrector components.

Once these issues are addressed, it may be feasible to even further extend the pulse approach to take the effect of powder layer on the temperature field into consideration. In the context of continuum thermal modelling of PBF-LB/M, the powder layer is often modelled via reducing the thermal conductivity of respective elements and increasing the absorption of laser power over the affected region. These considerations can be treated as inhomogeneous material properties, and accounted for via a combination of above-mentioned strategies.

Lastly, the presented networks are only suited to predict the temperature profiles in response to a constant laser power and scanning speed. It may be possible to train the pulse networks based on FE simulation results for scanning using various process parameters to reach a parametric solution similar to [28]. Such a surrogate model would be particularly useful in rapid evaluations of peak temperatures for a target geometry, and could help in optimising the process parameters accordingly.

## Conclusions

In this work, we presented a surrogate model of the heat transfer behaviour during the PBF-LB/M, where the continuously moving heat source is treated as a series of short laser scan segments called pulse events. First, the thermal response of pulses was represented by an FFNN, and trained based on FE simulation results for small scan patterns using constant material properties. This concept was initially demonstrated for the basic state of laser scanning on a build plate, and later extended to the case of a simple cuboid structure by parametrising the geometry effects. Ultimately, the base part of the framework was tested with temperature-dependent material properties, where temperature predictions remained accurate with considerable gains in computational performance.

Throughout this work, physical insights about the process could be exploited to help us reach a more general surrogate model that can provide accurate results for larger scan patterns that were not seen by the networks during training. For example, the symmetry in thermal response to single track laser scanning could be defined as a constraint on the input space coordinate and the introduction of a symmetry loss term. Another instance is the waning influence of the corners of a cuboid on laser scanning in the middle of it, that helped us reach a general solution for this type of geometry by only training the pulse corrector based on small scan area FE simulations. These results demonstrate the benefits of problem formulation with keeping the physical knowledge in mind as a bridge between unsupervised PINNs and fully supervised labelled training of neural networks.

In terms of computational benefits, an ML-based surrogate can offer tremendous gains. For example, 5 orders of magnitude faster evaluation time was observed in representing a nodal temperature history under a 15$\times$8 mm scan area versus conducting the FE simulation with temperature-dependent material properties. It is important to acknowledge that the development of a surrogate model has additional costs for running the small scale training FE simulations, and optimising the neural networks themselves. In addition, a trained surrogate is strictly limited to its process conditions during training, such as the scanning speed, or the laser power. However, in scenarios where numerous FE simulation using similar process conditions must be conducted for different scan patterns, these model offer the highest benefits. Furthermore, the evaluation times in the pulse approach remain independent of the complexities of the material model, while the FE simulation times substantially increase by introducing temperature dependence.

In conclusion, this work demonstrates the feasibility of the pulse approach in setting up a surrogate model for the heat transfer process in PBF-LB/M. There remain a number of hurdles to overcome for extending this framework to more general geometries, and more advanced materials in the future.

## Figures and Tables

**Fig. 12:**
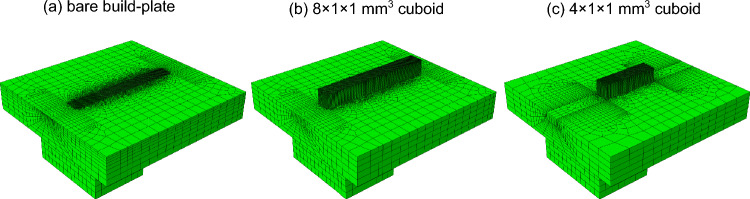
Various model geometries and their mesh distribution used in this study. **a** The bare build plate used for training and testing of the base function, representative of laser scanning over a wide solid block. **b** The cuboid used in setup and assessment of the corrector function. **c** A shorter cuboid used for generating training data. The meshes were generated using Abaqus CAE

**Fig. 13:**
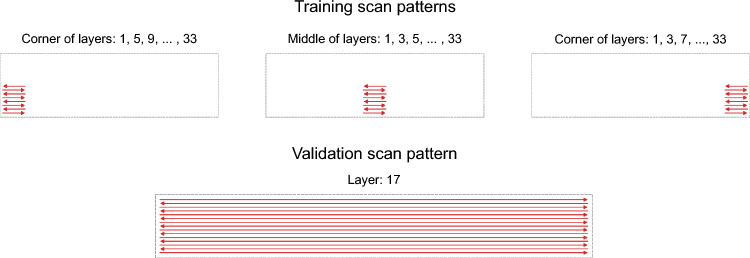
Example scan patterns used for training the geometry corrector neural networks compared to the reference scan pattern. The training set involved FE simulation in the middle and corner of cuboids for every other layer up to 1 mm build height (33 layers with a thickness of ${30}\,\upmu \text {m}$)

## Data Availability

The models and scripts used in this study are available under https://github.com/HighTempIntegrity/PIAM_Pulse2024.

## Appendix A: Finite element thermal simulations

This study used the Abaqus finite element (FE) software package to setup and run continuum thermal simulations for training and testing the presented neural networks. Below, the setup of the FE modelling framework is outlined first. Then, the model geometries and scan patterns used in the context of base and corrector functions are presented.

A common assumption in heat transfer analysis of PBF-LB/M involves representing the powder particles as continuum layers, and ignoring the phenomena related to liquid formation and motion [11, 13, 30]. Thereby, heat transfer in the continuum medium is governed by the Fourier equation:

A1 $$\begin{aligned} \rho c_p {\dot{T}} - \nabla \cdot (k\nabla T) = q_{\text {vol}} \end{aligned}$$

In above equation, $\rho$, $c_p$, and *k* are the density, specific heat capacity, and thermal conductivity of the material, respectively, and $q_{\text {vol}}$ represents heat generation from the moving laser spot. For most of the conducted simulations, the material properties were assumed to be constant, and equal to the values provided in Table 3. The heat source was implemented using the ‘toolpath-mesh intersection module’ of Abaqus based on the widely adopted Goldak formulation [31] as:

A2 $$\begin{aligned} q_{\text {vol}}=\eta \frac{6\sqrt{3}f_{f/r}P}{abc\pi \sqrt{\pi }}\exp \left( -3(\frac{x_m^2}{a^2_{f/r}}+\frac{y_m^2}{b^2}+\frac{z_m^2}{c^2})\right) \end{aligned}$$

Here, $[x_m,y_m,z_m]^T$ are the spatial coordinates in a moving Cartesian system attached to the laser centre, $\eta$ and *P* are the laser absorption coefficient and power, respectively, and ($a_{f/r}$,*b*,*c*,$f_{f/r}$) are geometrical parameters of the laser intensity distribution, which were assigned according to Table 3.

The above equations were solved using the Abaqus/Standard algorithm for various scan patterns. The solver offers an automatic time incrementation scheme that ensure the maximum residual heat flux and maximum nodal temperature corrections remain below a 0.5 % and 1 % threshold, respectively. This resulted in an average time increment duration of $1.14\times 10^{-4}\,\text {s}$ during laser scanning in the simulations. Layer addition was implemented based on the ‘quiet element’ approach where all model elements were present in the stiffness matrix from the start of the simulations, but inactive layers did not contribute to the solution. The simulations started with a uniform ${25}^{\circ }\text {C}$ temperature distribution over the whole domain, and a constant ${25}^{\circ }\text {C}$ boundary condition was applied at the bottom of the build plate stem.

**Table 3:** Model parameters used for setting up the FE simulations

Property	Value
Laser power (W)	300
Absorption coefficient $\eta$ (%)	26
Hatch distance ($\upmu \text {m}$)	70
Scanning speed ($\hbox {mm\,s}^{-1}$)	700
Recoating duration (s)	13
Scanning strategy	Bidirectional
Material properties	
Thermal conductivity ($\hbox {W}\,\hbox {m}^{-1}\,\hbox {K}^{-1}$)	50
Specific heat capacity ($\hbox {J}\,\hbox {kg}^{-1}\,\hbox {K}^{-1}$)	486
Density ($\hbox {kg}\,\hbox {m}^{-3}$)	8220
Goldak parameters	
$a_f$ ($\upmu \hbox {m}$)	60
$a_r$ ($\upmu \hbox {m}$)	240
*b* ($\upmu \hbox {m}$)	40
*c* ($\upmu \hbox {m}$)	60
$f_f$ (–)	0.4
$f_r$ (–)	1.6

Three types of geometries were used in the simulations as depicted in Fig. 12. In all scenarios, Abaqus CAE was used to mesh the models with an element size of $40\,\upmu \hbox {m}\,\times \,35\,\upmu \hbox {m}\,\times 30\,\upmu \hbox {m}$ in the laser interaction region based on convergence of the temperature field, while larger elements were used in the rest of the domain. Figure 12a shows the $12\,\text {mm}\,\times \,12\,\text {mm}\,\times \,2\text {mm}$ build plate, which accommodated various scan patterns shown in Fig. 4 used in training and testing of the base function. The target cuboid structure for development of the corrector function is shown in Fig. 12b. To generate the training data for small scan areas in the middle of the section, the same geometry was used. However, for the portion of training data related to scanning on the corners of the geometry, a smaller cuboid could be adopted (Fig. 12c), since the required correction to the base function in the far-field regions was negligible. Figure 13 shows a visual comparison of the training scan patterns in the middle and corners of the cuboids, and the full layer scan used for testing network performance.
